# Design of Electrohydrodynamic Devices with Consideration of Electrostatic Energy

**DOI:** 10.34133/2021/5158282

**Published:** 2021-01-09

**Authors:** Tasuku Sato, Shinya Sakuma, Masato Hijikuro, Shingo Maeda, Masayuki Anyoji, Yoko Yamanishi

**Affiliations:** ^1^Kyushu University, 744 Motooka, Nishi-ku, Fukuoka 819-0395, Japan; ^2^Kyushu University, 6-1 Kasuga Koen, Kasuga-shi, Fukuoka 816-8580, Japan; ^3^Shibaura Institute of Technology, 3-7-5 Toyosu, Koto-ku, Tokyo 135-8548, Japan

## Abstract

The importance of actuators that can be integrated with flexible robot structures and mechanisms has increased in recent years with the advance of soft robotics. In particular, electrohydrodynamic (EHD) actuators, which have expandable integrability to adapt to the flexible motion of soft robots, have received much attention in the field of soft robotics. Studies have deepened the understanding of steady states of EHD phenomena but nonsteady states are not well understood. We herein observe the development process of fluid in a microchannel adopting a Schlieren technique with the aid of a high-speed camera. In addition, we analyze the behavior of fluid flow in a microchannel that is designed to have pairs of parallel plate electrodes adopting a computational fluid dynamics technique. Results indicate the importance of considering flow generated by electrostatic energy, which tends to be ignored in constructing and evaluating EHD devices, and by the body force generated by the ion-drag force. By considering these effects, we estimate the development process of EHD flow and confirm the importance of considering the generation of vortices and their interactions inside the microchannel during the development of EHD devices.

## 1. Introduction

The importance of actuators, which can be integrated with flexible robot structures and mechanisms, has increased as soft robotics has become more advanced [1–4]. Soft materials, such as gels, papers, fluids, and biomaterials, have been actively studied to construct soft robots in efforts to mimic distinctive biological functions of living organisms [5, 6]. Soft actuators should not only mimic biological features but also realize functions of bioinspired robots superior to those of living organisms [7, 8]. The actuation mechanism of soft actuators differs from that of conventional rigid actuators, and design strategies must therefore be discussed prior to the integration of soft actuators into soft robotic systems [9, 10].

Fluid actuators, such as hydraulic and pneumatic pressure actuators, have been widely investigated. However, soft robots are difficult to control digitally because their components bend easily. The motion of a fluid actuator changes continuously, and the fluid actuator is thus suitable as a power source for soft robotics [11, 12]. Furthermore, fluid actuators can have high-speed responses on the millisecond order and, depending on the driving pressure, a larger force per volume than polymer actuators [13] when fluid paths are supported by tubes [14]. Although fluid actuators have remarkable compatibility with soft robots, there is currently a limitation when constructing a robotic system. Typically, the driving source is a pumping system that is much larger than the actuator. Therefore, an alternative driving source with a size comparable to that of the actuator is highly desired.

One alternative driving source of fluid actuators may be electrohydrodynamic (EHD) devices. In EHD phenomena, dielectric fluids or electrically charged fluids move isotopically depending on the applied voltage. Their movement is represented by electroosmotic flow and dielectrophoresis [15, 16]. EHD phenomena can be induced by simply applying a voltage to the fluid, and they can thus serve as a driving source through the integration of electrodes into a microfluid circuit. In addition, an EHD phenomenon has low power consumption, which should help downsize the overall system. Several EHD devices have been proposed in previous works. These works indicated that breaking the structural or electrical symmetry is important in extracting useful work as an EHD pump from isotopic phenomena [17, 18]. These studies demonstrated the usefulness of EHD phenomena for an on-chip pumping source but did not discuss details of the design parameters owing to difficulties in observing high-speed phenomena.

We herein construct an observation system adopting a Schlieren technique with the aid of a high-speed camera. We investigate the behavior of fluid flow in the microchannel of an EHD actuator based on electrostatic energy, which is typically ignored when constructing and evaluating EHD devices, and the body force generated by the ion-drag force. In addition, we analyze the development process of an EHD fluid in a microchannel adopting computational fluid dynamics (CFD). Finally, we discuss basic design strategies of EHD devices to obtain one-way flow.

## 2. Fundamental Equations of Electrohydrodynamics

As originally reported by J.A. Stratton in 1941 [19], the body force producing an EHD phenomenon **f**_**e**_ is expressed as

(1) $$\begin{array}{c} f_{e}=\rho_{e}E-\frac{1}{2}E^{2}\nabla \varepsilon +\frac{1}{2}\nabla \left[E^{2}{\left(\frac{\partial \varepsilon }{\partial \rho }\right)}_{T}\rho \right], \end{array}$$

where *ε*_0_, *ε*_*r*_, *ρ*_*e*_, *ρ*, and **E** are, respectively, the permittivity of a vacuum, relative permittivity, charge density, dielectric liquid density, and electrical field. Equation (1) was formulated on the basis of the electrostatic energy generated by applying an electrostatic field to a dielectric fluid. Only the second and third terms of Eq. (1) were derived from the electrostatic energy equation. The first term was added as a correction to express the electrophoretic force, which is a force generated when a dielectric fluid contains an ionic species or charged particle. The EHD mechanism in previous research is based on an ion-drag force. Applying a high voltage to a neutral molecule dissolves the molecule into its ionic species. The ionic species is accelerated by the electric field to generate an ion-drag force [20, 21].

Additionally, the terms in Eq. (1) represent different forces. The first term expresses the body force due to the ion-drag force generated by the conversion of a neutral species into an ionic species through the injection of charge. Although the ion-drag force can generate a high pressure, it can also severely deteriorate fluids. Hence, breakdown may readily occur. Moreover, a higher power supply frequency corresponds to less generated pressure. The second term expresses the dielectrophoretic force. Although the amount of charge injected has a small effect, a nonuniform electric field and nonuniform dielectric constant due to the temperature gradient affect the generated force. The third term expresses the electrostriction force, which is the strain induced when applying an electric field to a dielectric body. This force is affected by both spatial changes in the electric field and changes in the liquid temperature.

The applicability of Eq. (1) to EHD phenomena has previously been investigated. In 1991, Eq. (1) was introduced to describe EHD phenomena and expresses the general force generated when an electric field is applied to a dielectric fluid [22]. In the 2000s, EHD phenomena in a small space were studied to improve efficiency [23]. Ion drag has been reported to be the driving force of electrohydrodynamics [24]. Herein, we only consider the equation established in 1959 to express the ion-drag pressure [25] and assert that the first term in Eq. (1) has the greatest effect on EHD phenomena. Furthermore, in 2005, a study coupled electrohydrodynamics and hydrodynamics by combining macroscale and microscale flows [26].

In 2011, in-channel observations of electrohydrodynamics revealed the presence of vortices in a channel [27]. If only the ion-drag force is considered, vortices generated in the direction opposite the electric field cannot be explained. According to the first term in Eq. (1), the direction of fluid flow depends only on the electric field. Hence, the cited study's explanation of the principle of EHD flow with a conceptual picture of a kind of wall jet remains controversial. In addition, the cited study speculated that the dominant force in EHD phenomena is the Coulomb force by citing a 2003 paper written by Jeong et al. They made three assumptions: (1) the state is steady, (2) the fluid is incompressible and a single phase, and (3) the fluid is static. By making these assumptions, they ignored the second and third terms in Eq. (1) and only considered the electrophoretic force in calculating pressure [28]. Consequently, the second and third terms, which are derived from the electrostatic energy, become meaningless. Only the first term, which is a correction not formulated from calculation results, is useful.

When a device is developed using electrohydrodynamics, the experimentally observed pressure does not follow the square law written as Eq. (1) even if the resistance of the flow path is considered [29]. Although some EHD devices have been designed using Eq. (1), it is problematic that only EHD devices with parallel plate electrodes can be designed with this equation alone. Considering the above issues, how electrohydrodynamics is generated must be elucidated. In this study, we investigate the behavior of fluid flow in the microchannel of an EHD actuator and discuss basic design strategies based on Eq. (1).

## 3. Materials and Methods

### 3.1. System for Observing Electrohydrodynamics

**Figure 1:**
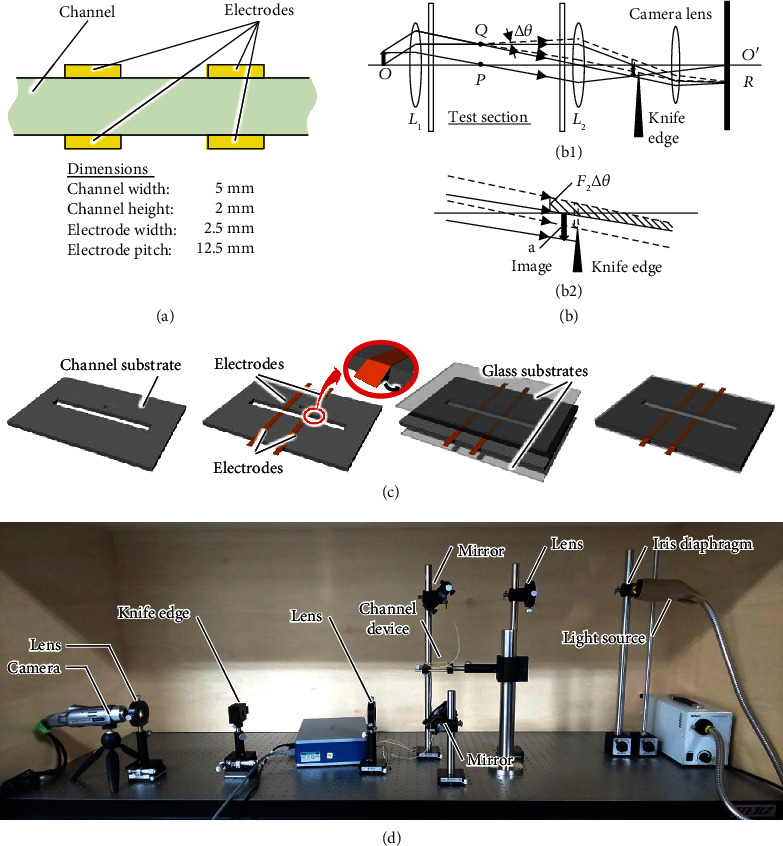
Experimental setup of the visualization system: (a) design of the EHD device, (b) schematic of the optical system where (b-1) shows the overall optical system and (b-2) is an enlarged view around the knife edge, (c) process of fabricating the EHD device, and (d) photograph of the constructed visualization system integrated with the fabricated EHD device.

To simplify the discussion, we chose a two-dimensional electrode arrangement where two different fields are in contact.  Figure 1(a) illustrates the device design with a pair of parallel plate electrodes. The flow profile developed in a microchannel of an EHD device. To visualize EHD phenomena, we adopted the Schlieren technique, which is an optical method that highlights differences in local density (Figure 1(b)). When optical turbulence occurs at point *Q*, the rays passing through point *Q* are refracted. This affects the brightness of the image on the screen. The brightness change *∆I* depends on the change in the refractive index *∆θ* and can be expressed as

(2) $$\begin{array}{c} \frac{∆I}{I_{o}}=\frac{F_{2}∙∆\theta }{a}, \end{array}$$

where *I*_*o*_, *F*_2_, and *a* are, respectively, the original brightness, focal length of the rear lens, and minimum image size.  Figure 1(c) shows the fabrication process. The flow channel was fabricated using a three-dimensional printer (AGILISTA-3200, KEYENCE, Osaka, Japan) while the electrodes were arranged with copper tape (CU-35C, 3M, St. Paul Minnesota, USA). The top and bottom sides of the microchannel were packaged using glass substrates with a photo-curable resin (BONDIC, Spirit of Wonder, Tokyo, Japan).  Figure 1(d) shows the constructed observation system. The emitted light was used as a point light source with an iris diaphragm. Collimated light was produced by passing the light through an achromatic lens placed at the focal length. The EHD device was installed in the position of the “channel device” shown in Figure 1(d). The collimated light was recondensed by an achromatic lens, and the main light flux was cut using a knife edge at the focal length. The cut light was focused and imaged by a lens, so that it was focused at the focal point in the camera. A high-speed camera (HX-3, nac Image Technology, Tokyo, Japan) was installed in the optical circuit to visualize the high-speed EHD phenomenon.

### 3.2. Analysis of Developing EHD Flow

To evaluate the process of flow development using the obtained images, we adopted an optical flow technique based on the Horn–Schunck method. The method was originally derived from the spatial constriction of the transfer energy of brightness. However, it can be applied to describe the complex fluid development process to reach the turbulent phase. We herein calculated vector maps using the algorithm proposed by Liu and Shen [30] and MATLAB (R2015b, MathWorks, Natick, Massachusetts, USA). It is noted that two time intervals were employed by considering the response of the development process of EHD flow. There was fast local flow around the electrode in the early stage of flow development, and we therefore used a time interval of 0.5 ms against a time period up to 300 ms after applying a voltage. After 300 ms, there is a large difference between the flow in the vicinity of the electrodes and the flow covering the flow channel, which tends to cause calculation errors. To eliminate calculation errors, we used a time interval of 2.5 ms for the time period from 300 ms because fluid development was slower in this stage and one-way flow was achieved.

### 3.3. CFD Analysis of Electrohydrodynamics

We adopted CFD analysis to confirm the contribution of each term of Eq. (1) in the EHD flow. Assuming that Eq. (1) expresses an EHD flow, the only variable in this equation is the steady electric field generated by the input voltage. The equation is thus transformed so that all terms in Eq. (1) are retained. We use the Clausius–Mossotti relation to obtain functions of permittivity and density. The Clausius–Mossotti relation is written as

(3) $$\begin{array}{c} \frac{\varepsilon_{r}-1}{\varepsilon_{r}+2}\frac{M}{\rho }=\frac{N_{0}\alpha }{3\varepsilon_{0}}, \end{array}$$

where *M*, *N*_0_, and, *α* are, respectively, the molecular weight, Avogadro's number, and polarizability. When Eq. (3) is transformed into a function of permittivity and density, part of the third term of Eq. (1) can be expressed as Eq. (4) assuming that the temperature is constant:

(4) $$\begin{array}{c} \rho \frac{\partial \varepsilon }{\partial \rho }=\rho \varepsilon_{0}\frac{\partial \varepsilon_{r}}{\partial \rho }=-\frac{1}{3}\varepsilon_{0}\left(\varepsilon_{r}-1\right)\left(\varepsilon_{r}+2\right). \end{array}$$

From Eqs. (1) and (4), we obtain

(5) $$\begin{array}{c} f_{e}=\rho_{e}E-\frac{1}{2}\varepsilon_{0}\varepsilon_{r}\nabla E^{2}+\frac{1}{2}\nabla \left[E^{2}\left(-\frac{1}{3}\right)\varepsilon_{0}\left(\varepsilon_{r}-1\right)\left(\varepsilon_{r}+2\right)\right]. \end{array}$$

We performed CFD analyses by applying the physical properties of hydrofluoroether (Novec7300, 3M) to this equation. Hydrofluoroether is a solution that serves as a hydraulic solution for electrohydrodynamics. In the following sections, the parameters *ε*_0_, *ε*_*r*_, and *ρ* are set, respectively, as 8.85 × 10^−12^ F/m, 6.1, and 1660 kg/m^3^.

## 4. Results

### 4.1. Observation of Flow Development in Electrohydrodynamics

**Figure 2:**
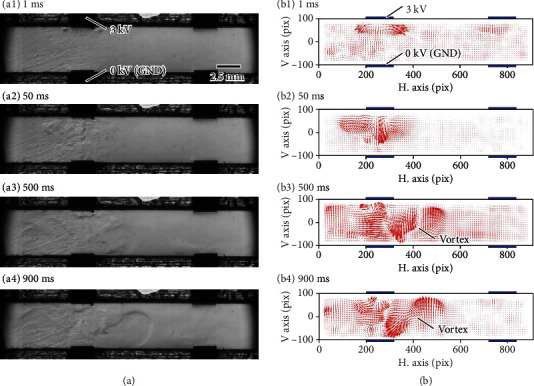
Experimental results obtained by activating the left-side electrodes. Photographs are extracted from Supplemental Movies S1 and S2. (a) Sequential images taken by the constructed visualization system. (b) Vector diagram obtained from image analysis using an optical flow where one pixel corresponds to 27 *μ*m. Blue bars indicate the positions of electrodes.

Figure 2 shows the visualization results of the flow obtained using the constructed observation system at an imaging speed of 2000 fps. The microchannel was filled with hydrofluoroether. Voltages of 3 and 0 kV were applied to the upper and lower electrodes on the left side, respectively. The right-side electrodes were not connected to the electrical circuit in this experiment. These conditions were set to target structural or electrical symmetry. An electric field was induced at the unconnected right-side electrode shown in Figure 2 and Supplemental Movie S1 but its effect was negligible.

Flow was generated at the left-upper electrodes where the electric field was strongest (Figure 2(a)). The flow became more complicated with time, with vortices forming beside the electrode. To clarify the process of flow development, we analyzed the obtained images adopting an optical flow-based image analysis technique proposed by Liu and Shen [30] (Figure 2(b)). We extracted a pair of images from the obtained video data and performed image analysis after masking unnecessary parts of the image. Local flow was generated from the edges of the left electrodes. The local fluid gathered and flowed toward the lower left (~50 ms). The gathering flow developed through the involvement of the surrounding fluid (~500 ms). Vortices thus formed in a microchannel (~900 ms). Although the vortices were positioned slightly off center relative to the electrodes, they maintained their relative positions during development. Additionally, there was no obvious net flow during the experiment. These results indicate that the assumptions of a steady state, an incompressible fluid in a single phase, and a static fluid are not applicable, even though they tend to be adopted in the development of EHD devices. Consequently, considering only the first term in Eq. (1) is inadequate for the design of an EHD device.

**Figure 3:**
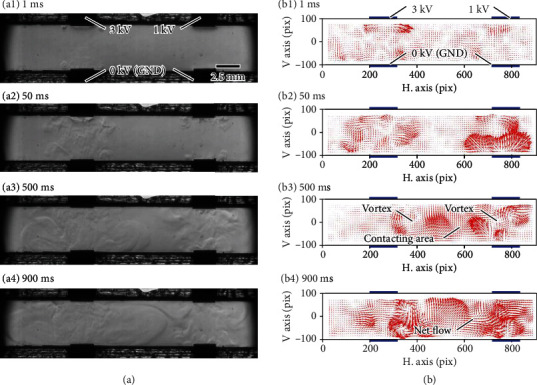
Experimental results obtained by activating electrodes on both sides. Photographs are extracted from Supplemental Movies S1 and S2. (a) Sequential images taken by the constructed visualization system. (b) Vector diagram obtained from image analysis using an optical flow where one pixel corresponds to 27 *μ*m. Blue bars indicate the positions of electrodes.

Figure 3 shows the visualization results when voltages of 1 and 0 kV were additionally applied to the upper and lower electrodes on the right side, respectively. The additional voltage was used to break the structural or electrical symmetry. Local flow was generated (~50 ms), and vortices then formed through the involvement of the surrounding fluid (~500 ms) around the electrode pairs (Figure 3(b)). The vortices on each side interfered with one another, generating a large vortex that covered the microchannel. It is noted that net flow occurred from left to right under this condition. The results also indicated that the EHD phenomena should be evaluated in terms of the time development. However, the time development cannot be obtained by considering only the first term in Eq. (1).

### 4.2. CFD Analysis for the Time Development of Electrohydrodynamics

**Figure 4:**
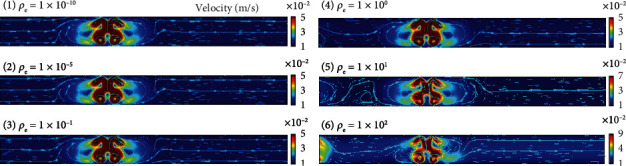
Results of finite element analysis obtained by varying *ρ*_*e*_ from 1 × 10^−10^ to 1 × 10^2^ where all terms in Eq. (5) are considered.

We first determined the charge density *ρ*_*e*_, which was an unknown parameter determined by Zhao and Adamiak [31]. We evaluated the effect of charge density on the flow profile in the developing stage by varying *ρ*_*e*_ from 1 × 10^−10^ to 1 × 10^2^.  Figure 4 shows the calculation results. The results were extracted 500 ms after the voltage was applied. It is noted that the pair of electrodes was activated with a voltage of 3 kV (left) in this simulation. All values of *ρ*_*e*_ had the same tendency for flow development. The second and third terms of Eq. (1) mainly affected the generation of vortices in the microchannel. However, the first term induced a force that interrupted the generated vortices and displaced the initial vortex positions along the direction of the electric field. As the vortex positions changed inside the microchannel, the interaction of the vortices generated by both electrodes of the pair changed. The interaction of the generated vortices generated a unidirectional flow from the high-voltage side to the low-voltage side. Hence, the charge density contributes to the intensity of the generated flow but not the flow profile. We determined *ρ*_*e*_ as 0.01 from these results.

We performed CFD analyses for two cases. In case 1, only the left electrodes were activated, with a voltage of 3 kV. This scenario allows the individual evaluation of each term in Eq. (5) and the confirmation of its contribution. In case 2, both electrodes were activated, with voltages of 3 kV (left) and 1 kV (right). The scenario allows confirmation that vortex development contributes to one-way flow for on-chip pumping.

**Figure 5:**
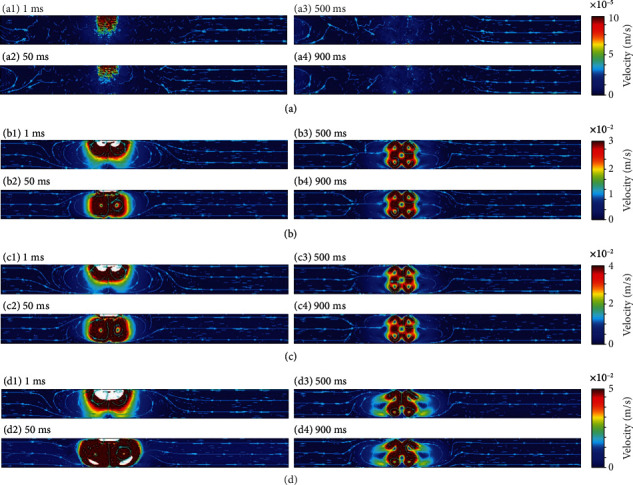
Results of finite element analysis obtained using Eq. (5) considering (a) only the first term representing the electrophoretic force, (b) only the second term representing the dielectrophoretic force, (c) only the third term representing the electrostrictive force, and (d) all terms in Eq. (5).

Figure 5 shows the simulation results for case 1, where the charge density *ρ*_*e*_ is 0.01 while the other parameters reflect the physical properties of the hydrofluoroether. The light blue lines in the CFD analysis show streamlines. Figure 5(a) shows the calculation results obtained using only the first term of Eq. (1). Only local flow was generated around the electrodes in the early stage of flow development. However, local flow disappeared with time, and eventually, a stationary flow formed in the steady state. Consequently, consideration of only the first term of Eq. (1) does not allow the simulation of flow development observed in Section 4.1. Figures 5(b) and 5(c) show the flow development leading to vortex generation when considering the second and third terms of Eq. (1), respectively. The light blue lines in the CFD analysis show the streamlines. Although the intensities of flow differed in these two cases, the flows formed around the electrodes and developed to induced continuous flow in the microchannel. Figure 5(d) shows the CFD result when all terms in Eq. (1) are considered. The flow profile was similar to those in Figures 5(b) and 5(c), demonstrating that the local flow around electrodes derived using the first term of Eq. (1) helps the development of vortices.

**Figure 6:**
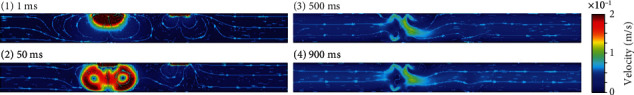
Results of finite element analysis where the left-side and right-side pairs of electrodes were activated by applying 3 and 1 kV, respectively.

Figure 6 shows the simulation results for case 2 obtained using the same physical parameters as used for case 1. The first term of Eq. (1) represents the force acting in the direction along the line of the electric force, and the generated flow was interrupted by vortices generated by the second and third terms of Eq. (1) near each electrode pair within 50 ms. The large vortex generated by the left pair of high-voltage electrodes merged with the small vortex generated by the right pair of low-voltage electrodes after 500 ms. The vortices interacted with each other and gradually formed a unidirectional flow from the high-voltage side to the low-voltage side. Consequently, all terms of Eq. (1) must be considered when designing EHD devices.

## 5. Discussion

After the initial generation of fast local flow, a slowly flowing vortex that covers the flow channel develops (Figure 2). The generated vortices reach a turbulent phase inside the microchannel, and the generated flow thus cannot be assumed to be a steady state, an incompressible fluid in the single phase, or a static fluid. The results of CFD analysis (Figure 5) show that the fast local flow is a feature of the first term of Eq. (1), but it does not generate the vortices. Meanwhile, both the second and third terms of Eq. (1) contribute to the formation of slow vortices. In addition, vortices are generated inside the microchannel, which helps generate unidirectional flow (Figures 5 and 6). These results demonstrate that the development process of EHD flow cannot be estimated using only the first term of Eq. (1). Consequently, it is important to consider all terms in Eq. (1) in generating vortices and their interactions inside the microchannel during the development of EHD devices to obtain one-way flow.

## 6. Conclusion

We herein investigated the behavior of EHD flow in a microchannel. Although the fundamental equation comprises three terms where two terms are based on electrostatic energy and the other is introduced as a correction for the body force due to the ion-drag force, researchers tend to ignore the electrostatic energy terms when constructing and evaluating EHD devices owing to difficulties in describing the development of the flow profile. We estimated flow development using observations made adopting a Schlieren technique and CFD analysis to obtain one-way flow. On the basis of these results, we discussed basic design strategies for the development of EHD devices. By appropriately designing vortex generation, fluid actuators can be realized with EHD pumps using a unidirectional flow.

## Data Availability

The MP4.movie data used to support the findings of this study are included within the supplementary information files.

## Supplementary Materials

Supplementary 1 Supplemental movie S1. Multimedia video file S1 shows the observation results. Activating (a) the left-side electrodes and (b) both the left- and right-side electrodes.

Supplementary 2 Supplemental movie S2. Multimedia video file S2 shows the analyzed vector diagrams. Activating (a) the left-side electrodes and (b) both the left- and right-side electrodes.
